# Game-Based Digital Interventions for Enhancing Positive Development and Addressing Substance Use in Adolescents: A Systematic Review

**DOI:** 10.3390/children11121554

**Published:** 2024-12-21

**Authors:** Serim Lee, Jinyung Kim, Sarah Bockhold, Jimin Lee, JongSerl Chun, Mansoo Yu

**Affiliations:** 1 Department of Social Welfare, Ewha Womans University, Seoul 03760, Republic of Korea; serimlee@ewha.ac.kr (S.L.); jiminlee28@ewhain.net (J.L.); jschun@ewha.ac.kr (J.C.); 2 School of Social Work, University of Maryland, Baltimore, MD 20742, USA; jinyung.kim@ssw.umaryland.edu; 3 School of Social Work, University of Missouri, Columbia, MO 65211, USA; sklwbc@umsystem.edu; 4 Department of Public Health, University of Missouri, Columbia, MO 65211, USA

**Keywords:** game-based intervention, digital game, positive development, substance use, adolescents

## Abstract

This study systematically reviewed research on the use of digital game approaches for the prevention, assessment, and treatment of substance use and positive development in adolescents. **Background/Objectives**: This study aimed to identify and summarize gaps in the published literature on game-based digital interventions for substance use and positive development for adolescents through a systematic review. **Methods**: Following PRISMA guidelines, 26 studies were selected for final analysis from an initial screening of 1601 references. Data were coded for multiple components, including study characteristics, intervention types, intervention contents, sample characteristics, substance use, positive development details, measurement tools, and main findings, particularly emphasizing the effectiveness of game-based digital interventions and the facilitators and barriers affecting program effectiveness. **Results**: Regarding substance use, 50% of studies reported game-based digital interventions as effective in reducing substance use, 16.7% as partially effective, and 33.3% as not effective. For attitudes toward substance use, 65.0% demonstrated statistical effectiveness, 25.0% indicated partial effectiveness, and 10% found no significance. Among studies that included a positive development component, 35.7% were effective, 50% were partially effective, and 14.3% were not effective. Very few studies have explored the facilitators and barriers to program effectiveness without statistical verification. **Conclusions**: This study offers valuable insights into the development and validation of game-based digital interventions, thereby establishing a robust foundation for their efficacy.

## 1. Introduction

A game-based intervention is a type of approach that incorporates games into existing services [1]. While games are fun and captivating, game-based interventions differ from purely recreational games in that their core purpose is to provide motivation and education [2]. This approach has increasingly been recognized as an effective and promising strategy for promoting attitudinal and behavioral changes [1]. As interest in game-based digital interventions continues to rise, a growing body of research has investigated their specific impact on mental health disorders [3], attitudes toward substance use [4], and positive adolescent development [5], with positive outcomes documented in each area.

According to self-determination theory (SDT), engaging in digital games can fulfill users’ psychological needs for competence, relatedness, and autonomy [6]. Consequently, digital games designed for interventions offer distinct advantages, such as improving user accessibility, increasing engagement, and reducing stigma [7]. Since gaming is highly popular among teens, digital environments and gamified approaches offer new opportunities for promoting health, especially in the case of adolescents [8]. On average, adolescent boys and girls spend approximately 120 and 40 min per day playing video games, respectively. 95% of adolescents aged 13–17 have access to a smartphone, 45% use the Internet almost constantly, 88% have access to a computer at home, and 90% engage in gaming on computers, phones, or gaming consoles [1]. Considering these factors, it is evident that game-based technologies and communication strategies significantly influence how adolescents learn and perceive health-related risks and protective behaviors [1].

Drug use among adolescents remains one of the most serious social problems in the United States. The percentage of adolescents who have engaged in illicit drug use exceeds 27%, although this percentage has significantly decreased since the pandemic [9]. Substance use in adolescence can lead to significant long-term morbidity and mortality [10]. Therefore, addressing substance use is crucial for ensuring the healthy development of adolescents. Generally, perceptions regarding peers’ drug use tend to align closely with actual usage levels. There are strong correlations at the individual level between drug use and attitudes or beliefs regarding that substance [9]. Individuals who perceive that using a specific drug poses a risk of harm or who disapprove of its use are less likely to engage in that behavior [9].

Positive development fundamentally outlines the components of ideal adolescent development and provides opportunities for youths to enhance their strengths. Most importantly, it plays a significant role in preventing substance use among adolescents [11]. A study that developed an Integrated Positive Youth Development framework for preventing adolescent drug use identified 16 key constructs, including promoting bonding, resilience, social and emotional competence, cognitive and behavioral competence, moral competence, self-determination, self-efficacy, spirituality, positive future beliefs, identity development, recognition of positive behavior, opportunities for prosocial involvement, prosocial norms, and money literacy [11]. Self-efficacy, one of the 16 components of positive development, predicts smoking behavior during adolescence and serves as a protective factor that can regulate smoking behavior [12,13]. In this way, the elements of positive development directly influence adolescents’ delinquent behaviors. Therefore, it is recommended that these components be actively supported and addressed to effectively prevent and mitigate specific behaviors among adolescents [11,13].

Game-based digital interventions are actively employed as a strategy to enhance the positive development of adolescents. These interventions have generally demonstrated positive effects on adolescents’ self-efficacy and various other components of positive development, including social competence, behavioral competence, cognitive competence, and academic achievement [14,15,16,17]. Adolescence presents a prime period for addressing adverse health behaviors given their cognitive flexibility, prosocial growth [18], and transition into adulthood, which is accompanied by multiple physical and psychological changes [19]. In 1904, G. Stanley Hall discussed the “storm and stress” of adolescents navigating “the problematic interplay between low self-control (the “storm”) and increased vulnerability (the “stress”)”. This combination can lead to increased risk-taking behaviors, succumbing to peer pressures [18], and increased mental health needs [20]. Interventions for youths are needed for the treatment of substance use, as well as preventative interventions designed to educate, empower, and increase negative attitudes towards substance use. Addressing prosocial behavior is important to both positive development as well as central to improving the health of our youth and reducing risky behaviors such as substance use [18].

As technology continues to advance, so do game designs and constructs, introducing opportunities for developing self-efficacy to refuse opioids, reduce craving levels, and increase negative attitudes towards substance use through body motion and voice recognition technology in games such as “Recovery Warrior” while actively engaged in outpatient treatment for opioid use [21]. Other games are less costly and embed computer-tailored interventions into a serious game format to utilize the I-Change model focused on positive development and decreasing binge drinking in adolescents through the game Alcohol Alert [22]. The I-Change Model is based upon several theories and models, including the Attitude–Social Influence–Self-Efficacy Model, Theory of Reasoned Action, Theory of Planned Behavior, Social Cognitive Theory, Health Belief Model, Precaution Adoption Model, and Transtheoretical Model [22]. The model attempts to explain motivation and behavioral changes and includes such concepts as attitude, modeling, social norms, perceived pressure, and self-efficacy [22]. Still others have been designed to increase knowledge of tobacco marketing towards youths [23] or decrease tobacco use through playing “ACTION” and learning to reflect upon a variety of pressures youths are faced with in their community [24]. EmpathyAR was designed in an effort to create attractive experiences to develop empathy and prosocial behaviors utilizing augmented reality and geolocation [25].

Adolescent substance use treatment has appeared to be simply recycled from adult interventions [18], and more research is needed to better cater to a generation steeped in technology and developmentally distinctive in their physical, emotional, and cognitive presentation. It is important that these interventions are culturally tailored to populations of interest, as this review demonstrates a largely Western dominance in the current body of game-based digital interventions for youth substance use and positive development. Adolescents require interventions that aid in developing, defining, and maintaining healthy habits and which entice and sustain attention through a familiar vehicle such as gaming and technology [19]. The use of gamification, rather the process of engaging a learner through the incorporation of education and game elements such as challenges, goals, feedback, and rewards to promote positive health change and to address health issues within adolescents has been shown to be a promising intervention. This relaxed learning method has been found to be agreeable with adolescents and helps maintain their active engagement in learning [19,22]. The positive impact gamification can have on learning outcomes related to knowledge of and attitudes towards substance use, the treatment of substance use, and the development of prosocial behaviors involved with positive development is explored most within the theoretical constructs of such theories as Social Cognitive Theory (SCT), Theory of Planned Behavior (TPB), and Theory of Reasoned Action (TRA). Several game-based digital interventions such as “Recovery Warrior” suggest promising participant response (e.g., “all participants recommended playing the videogame as part of treatment at least weekly.”) [21], measuring the mechanisms at work remains an area of additional research [19]. More research is also needed to better understand the high attrition rates from programs despite positive responses to satisfaction surveys, suggesting adolescents’ preference for this method of intervention over traditional learning [1]. This study aims to identify and summarize gaps in existing research by conducting a systematic review examining the use of game-based digital interventions designed for positive development of adolescents as both treatment for substance use and prevention efforts to develop knowledge of and increase negative attitudes towards substance use.

## 2. Materials and Methods

### 2.1. Search Strategy

This systematic review comprehensively examined records from four databases—PubMed, PsycINFO, Scopus, and CINAHL—up to 6 September 2024, marking the initiation of the study period. No specific start date was imposed for article inclusion; therefore, all relevant articles, regardless of their publication date, were considered from the earliest available instance until 6 September 2024. These databases were selected based on their prevalent use in systematic reviews of similar research topics [26]. We utilized three sets of distinct topic keywords: (1) game(s) OR gaming OR mobile game OR video game OR active video OR digital game OR computer game OR online game OR serious gaming OR teleplay OR digital play OR exergame OR exergames OR exergaming OR gamified OR gamification OR virtual reality games OR VR games OR game-based OR game-based intervention [27,28,29,30], AND (2) substance use OR alcohol OR smoking OR cigarette OR e-cigarette OR HTPs OR drug OR cocaine OR marijuana OR opioid OR methamphetamine OR bonding OR resilience OR social competence OR emotional competence OR cognitive competence OR behavioral competence OR moral competence OR self-determination OR self-efficacy OR spirituality OR beliefs in the future OR positive identity OR prosocial involvement OR prosocial norms OR money literacy [11,31,32], AND (3) adolescent OR youth OR teenager OR middle school student OR high school student. We searched all fields in SCOPUS, the title and abstract fields in PubMed and PsycINFO, and the abstract field in CINAHL.

The misuse of drugs, including marijuana, opioids, cocaine, and methamphetamine, has been documented across various countries [31]. In the United States, the National Survey on Drug Use and Health (NSDUH) offers detailed information on substance use, encompassing marijuana, cocaine, heroin, methamphetamine, hallucinogens, inhalants, and prescription stimulant misuse [32]. For the positive development, a study that developed an Integrated Positive Youth Development Conceptual Framework for the prevention of psychotropic drug use among adolescents identified 16 positive youth development constructs: (1) promotion of bonding; (2) cultivation of resilience; (3) promotion of social competence; (4) promotion of emotional competence; (5) promotion of cognitive competence; (6) promotion of behavioral competence; (7) promotion of moral competence; (8) cultivation of self-determination; (9) development of self-efficacy; (10) promotion of spirituality; (11) promotion of beliefs in the future; (12) development of clear and positive identity; (13) recognition for positive behavior; (14) providing opportunities for prosocial involvement; (15) fostering prosocial norms; (16) promotion of money literacy [11].

### 2.2. Study Selection

In accordance with the PRISMA guidelines, this study was conducted in several stages: identification, screening (including eligibility assessment), and inclusion [33]. A total of 1601 references from each database were imported into Covidence (https://www.covidence.org/) [34], which automatically eliminated 388 duplicates, resulting in 1213 records for title and abstract screening. Two of the six reviewers conducted database searches using predefined keywords and imported the results into Covidence, while the remaining reviewers provided oversight throughout the process.

**Figure 1:**
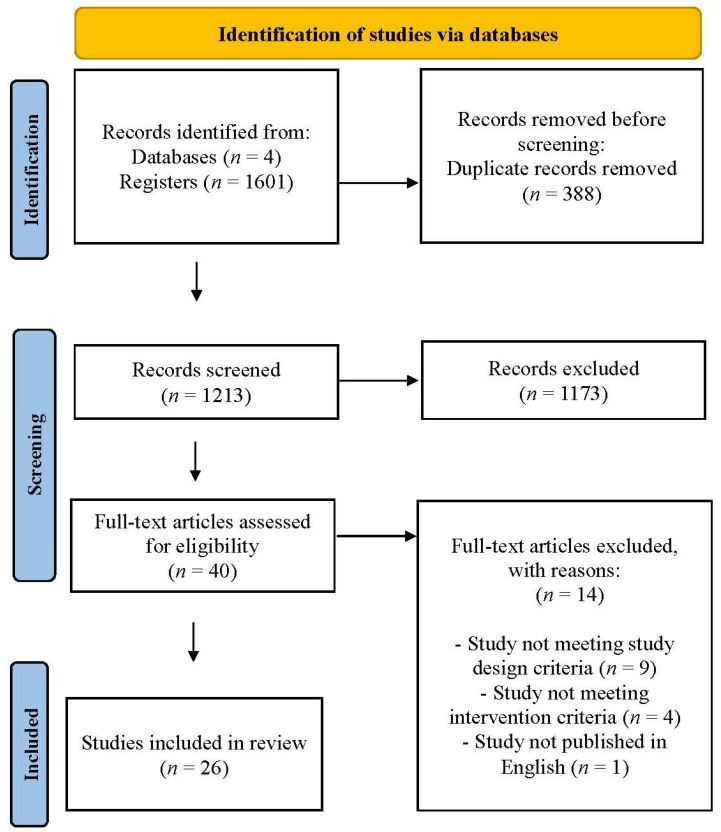
PRISMA flow chart.

This systematic review included studies that met predefined inclusion and exclusion criteria (See Table 1). The inclusion criteria required that studies (1) be peer-reviewed articles published in English regardless of the country where the studies were conducted, (2) contain any type of digital game-based intervention for substance use or positive development, (3) include randomized controlled trials (RCTs) and non-RCTs that investigated the effects of game-based interventions on substance use and positive development among adolescents, (4) provide all necessary data information (e.g., sample size, odds ratio, 95% CI, or other effect size values), and (5) be rated as “fair” or “good” based on the National Institute of Health (NIH) quality assessment tool [35]. Conversely, exclusion criteria encompass studies that are (1) master’s theses or doctoral dissertations, (2) observational studies (e.g., cross-sectional studies, case-control studies, or (retrospective or prospective) cohort studies), (3) qualitative studies, (4) commentary and editorials, (5) review papers including systematic review and meta-analysis, and (6) unavailability of the full text. Two out of six reviewers independently assessed each article as “yes”, “no”, or “maybe” based on these criteria. Discrepancies in ratings were resolved through discussion to reach a consensus, with oversight provided by the other reviewers. From the first screening stage, 1173 irrelevant records were removed, resulting in 40 articles advancing to full-text review. The application of predetermined criteria resulted in the exclusion of 14 articles, leaving 26 articles for final analysis (See Figure 1).

### 2.3. Data Extraction and Analysis

Before the coding process commenced, approximately 10% of the final sample was randomly selected for double screening to ensure consistency among raters [36,37]. The Kappa coefficient, which measures interrater reliability, was calculated for the two raters and found to be 0.838, demonstrating a strong level of agreement. Any discrepancies in wording or interpretation were collectively discussed and resolved by the four authors. The coding process captured a range of details, including author and year, study type, data source, sampling methods, sample characteristics (e.g., size, age range, mean age, gender distribution, racial demographics), type of game-based intervention (e.g., assessment, prevention, treatment), contents of the intervention (e.g., theoretical framework, duration, session), type of substance use, type of positive development, measurement tools used, and main findings/outcomes, which include the effectiveness of game-based interventions on substance use and positive development, as well as the facilitators and barriers impacting their effectiveness.

## 3. Results

### 3.1. Characteristics of Study

**Figure 2:**
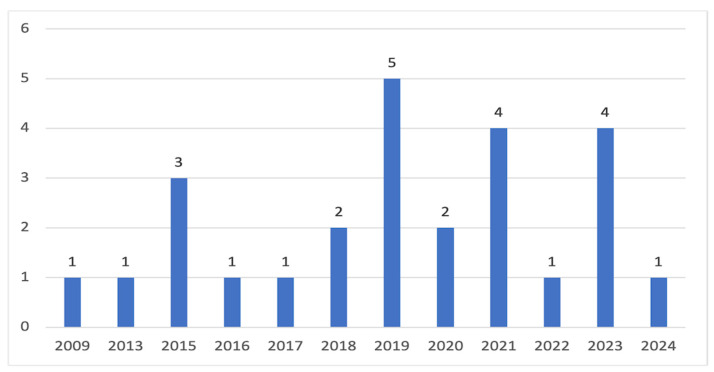
Number of studies by publication year.

The final sample of 26 studies (*n* = 26) was analyzed, with the results presented in Table A1. The publication year for these studies ranged from 2009 to 2024. As seen in Figure 2, the number of studies began to increase after 2018 and reached the highest number in the year 2019 (*n* = 5, 19.2%). Nearly half of these studies were conducted in the United States (*n* = 15, 57.7%), followed by the Netherlands (*n* = 3, 11.5%), Finland (*n* = 2, 7.7%), Australia (*n* = 2, 7.7%), Hong Kong (*n* = 1, 3.8%), Philippines (*n* = 1, 3.8%), Iran (*n* = 1, 3.8%), and Spain (*n* = 1, 3.8%). Of the 26 studies, 57.7% were RCTs (*n* = 15), while the remaining study types included 19.2% quasi-experimental (*n* = 5) and 23.1% pre-experimental (*n* = 6). In terms of the sampling methods, 46.2% implemented the convenient sampling method (*n* = 12), followed by cluster sampling (*n* = 8, 30.8%), stratified random sampling (*n* = 4, 15.4%), and purposive sampling (*n* = 1, 3.8%). Only two studies utilized a secondary dataset (7.7%), while the vast majority (*n* = 24, 92.3%) collected primary data.

### 3.2. Types and Contents of Game-Based Interventions

Of the twenty-six studies included in this study, fifteen (57.7%) were prevention programs, eight (30.8%) were treatment programs, and three (11.5%) were assessment programs. Eighteen indicated the specific applied theory/model/framework. Among these 18, 11 studies (61.1%) were the fusion of various theories, such as social cognitive theory (SCT), prospect theory, health behavior theory, reinforcement theory of motivation, attitude–social influence–self-efficacy model, theory of reasoned action (TRA), theory of planned behavior (TPB), health belief model (HBM), precaution adoption model, transtheoretical model/stages of change, psychosocial theory, information–motivation–behavioral skills model, game therapy, theory of gamified learning, and social learning theory (SLT). The SCT was the most applied theory, as eight out of eighteen (44.4%) applied it, followed by the TPB (*n* = 7, 38.9%) and the TRA (*n* = 3, 16.7%). Apart from the fusion of various theories, seven studies focused solely on a single theory, including the Communication Competence Model (CCM), self-efficacy theory, Cognitive Behavioral Game Design (CBGD) theory, and flow theory.

The studies included a range of digital gaming platforms, with 24 out of 26 (92.3%) specifying the techniques utilized for these games. Four studies (15.4%) focused on virtual reality (VR) and augmented reality (AR) games, including avatar-based virtual reality games and cyber–physical games based on augmented reality and geolocation. Five studies (19.2%) examined mobile games, which included social (team-based) mobile games, health-related mobile games, gamification-based prevention training mobile applications (including scoring, badges, rewards, and educational content), and interactive mobile games. Four studies (15.4%) addressed web-based games, including internet quiz games and online games. Additionally, three studies (11.5%) explored computer-based games, while three others (11.5%) focused on tablet-based games. Of these, two studies examined games that were playable on both computer and tablet platforms. Four studies (15.4%) were on video games, encompassing both web-based and tablet-based formats. Lastly, one study (3.9%) investigated digital games in general. The games were categorized by content type, including serious games (*n* = 4), interactive games (*n* = 2), avatar-based games (*n* = 2), educational games (*n* = 2), social games (*n* = 1), cognitive and behavioral games (*n* = 1), health-related games (*n* = 1), gamification and points-based games (n = 1), quiz games (*n* = 1), and other unique game concepts. 

A total of 25 studies (96.2%) provided information on the duration or timing of each intervention, with durations ranging from 1 day to 7 months. The most frequently reported duration was 4 weeks (*n* = 5), followed by 2 weeks (*n* = 3) and 1 day (including immediate interventions; *n* = 3). Other reported durations included 1–2 weeks, 5–7 days, 4 days, 6 weeks, and 7 months. The time allocated for each intervention varied widely, from as little as 2 to 5 min to as long as 4.5 h, with 30 min and 20–40 min being the most common durations, each cited by two studies. Of the studies, 22 reported the number of sessions, which ranged from one to six. The most frequently reported number was a single session (*n* = 7), followed by four sessions (*n* = 5) and two sessions (*n* = 3). Three studies indicated that the number of sessions varied depending on the participants’ needs, while individual studies reported three, five, six, and two to four sessions. The mean time participants spent engaging with the game-based intervention ranged from 10 min to 436.2 min, with an average of 86.4 min (*SD* = 115.8). 

Of the studies examining the period of cutting or reducing substance use following game-based interventions, six studies (23.1%) reported specific durations. The most frequently reported period was three months (*n* = 3), while one study reported durations of 90 days, four months, and four weeks, respectively. 

### 3.3. Sample Characteristics

The total sample size when combining all the studies resulted in 15,071 with a minimum value of six and a maximum value of 7792. The mean sample size was 579.65 (*SD* = 1560.67) and the median was 142. The mean age of the participants was 14.63 years old (*SD* = 2.44), ranging from 11 to 20.6 years of age. Meanwhile, the median age was 14.3 years old. With regards to the gender composition in the sample, approximately half of the studies had evenly distributed males and females in the sample (*n* = 15, 57.7%). Furthermore, 11.5% had a slightly higher female portion (*n* = 3), 7.7% had the majority of their sample as male (*n* = 2), 3.8% had a slightly higher male portion (*n* = 1), 3.8% had only females (*n* = 1), and 3.8% had only males (*n* = 1). 

### 3.4. Target

Among the 26 studies, eight (30.8%) focused solely on attitudes towards substance use; seven (26.9%) addressed both attitudes towards substance use and positive development; three (11.5%) targeted only positive development; three (11.5%) examined substance use, attitudes towards substance use, and positive development simultaneously; two (7.7%) focused exclusively on substance use; and two (7.7%) investigated both substance use and attitudes towards substance use. One study (3.9%) targeted both substance use and positive development together. That is, eight studies (30.8%) targeted substance use, 20 (76.9%) focused on attitudes towards substance use, and 14 (53.9%) addressed positive development. 

Regarding the eight studies that targeted substance use, four (50%) focused on alcohol, two (25%) on cigarettes (or tobacco), and two (25%) on opioids. Of the 20 studies that focused on attitudes towards substance use, five (25%) examined attitudes towards cigarettes (or tobacco), four (20%) targeted attitudes towards alcohol, four (20%) focused on attitudes towards opioids, and three (15%) explored attitudes towards various drugs including cannabis, ecstasy, methamphetamine, and hallucinogens. Two studies (10%) examined attitudes towards e-cigarettes, one study (5%) targeted attitudes towards both cigarettes and e-cigarettes, and one (5%) addressed attitudes towards general substance use. 

Among the 14 studies targeting positive adolescent development, eight (57.1%) focused on self-efficacy. Individual studies (7.1%) targeted social competence, behavioral competence, and academic achievement, respectively. Three studies (21.4%) explored multiple aspects of positive development: one examined empathy and prosocial behavior together; another targeted attitude, modeling, social norm, perceived pressure, and self-efficacy; and the third focused on cognitive reappraisal skills, beliefs/attitudes, self-efficacy, and emotional self-efficacy. 

### 3.5. Measurement Tools

For substance use, 26.9% (*n* = 7) of the studies measured frequency, which was adapted from other surveys, whereas only one study used a formal assessment tool such as the Alcohol Use Disorders Identification Test (AUDIT; Yap et al.) [38]. Meanwhile, attitudes towards substance use were assessed with adapted version of other tools such as the Youth Risk Behavior Survey (e.g., Weser, Duncan, Pendergrass et al. [39]; Weser, Duncan, Sands et al. [40]), Maryland Opioid Survey (e.g., Abraham et al. [5]; Abraham et al. [41]), Wisconsin Statewide Survey (e.g., Abraham et al. [5]; Abraham et al. [41]), National Youth Tobacco Survey (e.g., Hieftje et al. [42]; Pentz et al. [43]; Weser, Duncan, Sands et al. [40]), Legacy Media Tracking Survey (e.g., Gilliam et al. [23]) (*n* = 10, 38.5%), formal scales or questionnaires (e.g., Smoking Outcome Expect Scale [SOES]: Parisod et al. [44]), Attitudes toward Drinking and Alcoholism test ([SAADA]: Yap et al. [38], Penn alcohol craving scale [PACS]: Abroms et al. [21]; *n* = 5, 19.2%), questionnaires developed by the research teams in each study (e.g., Anti-Tobacco Industry Index [ATI]: Rath et al. [45]; *n* = 3, 11.5%), and unknown resources with just a Likert scale (*n* = 3, 11.5%). The review found that 23.1% (*n* = 6) of the studies used formal scales or questionnaires to measure positive development outcomes, such as the Anti-Smoking Self-Efficacy scale ([ASSES], Parisod et al. [44]), Brief Self-Report Scale ([BSCS], Boendermaker et al. [14]), Self-Efficacy Questionnaire for Children ([SEQ-C], Fernandes et al. [20]), Interpersonal Reactivity Index ([IRI], López-Faican & Jaen [25]), and Prosocial Behavior Questionnaire ([PBQ], López-Faican & Jaen [25]). Additionally, studies have also utilized tools adapted from other resources such as the Youth Risk Behavior Surveyand Medication Understanding and Use Self-Efficacy Scale ([MUSE], Abraham et al. [5]; *n* = 4, 15.4%), tools developed by the research team (*n* = 2, 7.7%), and unknown resources with a Likert scale (*n* = 1, 3.8%). 

### 3.6. Main Findings

#### 3.6.1. Effectiveness of Program—Descriptive Analysis

##### Substance Use

Regarding the eight studies [4,5,14,21,22,24,38,46] targeting substance use, two [21,46] presented descriptive analyses of the program’s effectiveness. One study [46], which focused on binge drinking, reported that participants in the game intervention group rated the intervention more favorably than those in the control group, who received a brochure (intervention *M*, 15.21 vs. control *M*, 13.49, *t*(124) = −2.50; *p* = 0.014). Another study [21] targeting opioid use, reported that, based on urine analysis, four out of nine participants (44.4%) remained abstinent from opioid use.

##### Attitudes Toward Substance Use

Among the twenty studies examining attitudes toward substance use, seven [1,16,39,40,42,44,47] reported descriptive results from satisfaction surveys concerning game-based interventions. Specifically, in relation to attitudes toward cigarettes, the game “Fume”, which targets tobacco-related health literacy, garnered significantly greater interest compared to the control group’s website (intervention, 70.0% vs. control, 27.7%; *p* < 0.001) [44]. Notably, 25.4% of participants described Fume as “very nice”, while 52.5% rated it as “nice” [44]. One study indicated that females were more likely to report enjoyment of the game (odds ratio [*OR*] 1.84; 95% *CI*, 1.27–2.66) and to indicate that they learned something new (*OR* 1.59; 95% *CI*, 1.06–2.38) compared to males, who were more likely to perceive the game as challenging (*OR* 4.11; 95% *CI*, 1.06–15.88) than participants who preferred to self-describe [42].

Participants engaging with the program aimed at influencing attitudes toward e-cigarettes reported high enjoyment scores (3.00 out of 4.00 in [39]; 3.08 out of 4.00 in [40]) and a sense of presence (2.91 out of 4.00) [40]. In the context of programs targeting attitudes toward drugs, 79% of participants indicated they learned new information, 88% found the experience enjoyable, and 97% reported ease of use [16]. Similarly, 91% expressed enjoyment of the game designed for attitudes toward illicit drugs, while 88% rated it as superior to a traditional booklet and 92% felt it made the lesson more engaging [47]. Lastly, regarding attitudes toward opioids, adolescents reported moderate to high levels of enjoyment for the game, although specific statistical data were not provided [1].

##### Positive Development

Among 14 studies that tested the effectiveness of gaming-based interventions on positive youth development, two [15,20] of them reported descriptive findings. Norris et al. reported that 3.33 out of four participants in the game intervention group liked the game, 90.5% felt it real, and 71.4% were deeply involved with it [15]. In the study by Fernandes et al., participants reported that the game was supportive (Mean [*M*] = 1.1), easy (*M* = 1.6), efficient (*M* = 1.1), and clear (*M* = 1.7) on a scale from −3 to 3 [20].

#### 3.6.2. Effectiveness of Program—Comparative Analysis

Table 2 presents a summary of the effectiveness of game-based intervention on substance use, attitudes toward substance use, and positive development.

##### Substance Use

Among the eight studies focused on substance use, seven [4,5,14,22,24,38,46] reported statistically significant effectiveness for game-based interventions, all of which targeted cigarette smoking or alcohol consumption. Of these, four [4,22,24,38] indicated that the interventions were “effective” in reducing substance use, one [46] reported them as “partially effective”, and two [5,14] concluded that they were “not effective”.

For cigarettes, group differences were significant at a 90-day follow-up for 3-day abstinence with verification (*β* (*SE*) = 1.49 (0.72); *p* = 0.048; *OR* 4.46; *CI*, 1.02–19.52) and 7-day abstinence with 3-day verification (*β* (*SE*) = 1.49 (0.72); *p* = 0.049; *OR* 4.44; *CI*, 1.01–19.49) [24]. Meanwhile, there was a significant increase in cigarette cessation “Time” (*F* (2, 138) = 79.50; *p* < 0.001; $\eta_{p}$^2^ = 0.54), but there was not a significant increase in “Dose” (*F* (1, 69) = 1.47; *p* = 0.230; $\eta_{p}$^2^ = 0.02) [46]. For drinking alcohol, significant results included reduced drinking behavior (risk ratio (*RR*) 0.79; 95% *CI*, 0.68–0.92; *p* = 0.003) [4], decreased alcohol consumption (*β* = −0.06, 95% *CI* = −0.11 to −0.01; *p* = 0.02) [4], fewer alcohol-related troubles (*β* = 0.62; 95% *CI*, 0.61 to 0.62; *p* < 0.001) [4], and decreased binge drinking both for 15-year-olds (after three sessions: *OR* 0.22; 95% *CI*, 0.09–0.54; *p* = 0.04) and for 16-year-olds after three sessions (*OR* 0.37; 95% *CI*, 0.16–0.87; *p* = 0.02) [22]. A significant interaction effect was observed between condition and educational level for excessive drinking (*OR* 2.37; 95% *CI*, 0.98–5.73; *p* = 0.05) among adolescents who adhered to at least one session. Adolescents reported less binge drinking in the previous 30 days if their parents also participated in the study (*p* = 0.04). Significant protective factors against binge drinking included having a higher educational level (*p* < 0.001), being younger (*p* < 0.001), and being Protestant (*p* = 0.03), Muslim (*p* < 0.001), or a member of another religion (*p* = 0.03). Similarly, significant protective factors against excessive drinking included being female (*p* < 0.001), having a higher educational level (*p* = 0.01), and being younger (*p* < 0.001) [22].

##### Attitudes Toward Substance Use

Among the twenty studies [1,4,5,8,16,17,21,22,23,38,39,40,41,42,43,44,45,47,48,49] targeting attitudes toward substance use, thirteen [4,5,16,17,23,38,41,42,43,44,45,48,49] reported statistical effectiveness, five [1,22,39,40,47] indicated partial statistical effectiveness, and two [8,21] found no statistical effectiveness. Regarding attitudes toward alcohol, the game intervention was significant in shaping the perceptions of parents and peers toward anti-alcohol attitudes (*β* = 0.06; 95% *CI*, 0.00–0.12; *p* < 0.05) and alcohol-related knowledge (*β* = 0.18; 95% *CI*, −0.01 to 0.36; *p* = 0.07) [4]. Similarly, the game intervention resulted in a significantly greater increase in alcohol knowledge (Cohen’s *d* = 2.54) compared to the video intervention (Cohen’s *d* = 0.89; *p* = 0.00) [38]. The longer participants played the game, the more positive their attitudes toward binge drinking became (*β* = 0.12; *p* < 0.05); conversely, higher scores in the game were associated with more negative attitudes toward binge drinking (*β* = −0.25; *p* < 0.001) [48].

Regarding attitudes toward cigarettes (or tobacco), studies generally reported significant effectiveness, including changes from pre- to post-gameplay in both positive (*p* = 0.002) and negative (*p* = 0.02) smoking outcome expectations within the intervention group [44]. A significant decrease in attitudes toward cigarette smoking (*p* = 0.01) was also reported within the intervention group, where a lower score indicates a more negative attitude toward cigarette smoking [44]. Similarly, the self-reported perceived knowledge of the health effects of tobacco significantly increased from pre- to post-gameplay (*M* = 2.81, vs. *M* = 3.31; *t* = −4.79; *p* < 0.001) [23]. Additionally, the level of mastery achieved in the game was significantly associated with an increase in the ATI Index (Attitudes about Tobacco Products and the Tobacco Industry) score at the 3-month follow-up (mean difference *MD* = 0.6, Cohen’s *d* = −0.36; *p* < 0.0001), where a higher score indicates a more negative attitude toward the tobacco industry [45]. Following the intervention, significant differences in the proportion of correct answers were observed for questions related to beliefs about the health harms of cigarettes and e-cigarettes, their danger, and the potential for teen addiction, as well as for questions assessing the knowledge of nicotine content, tobacco marketing toward teens, the impact on teen brain development, and the likelihood of smoking as an adult (all *p* < 0.0001) [42]. However, Nyman et al. reported no effectiveness in motivation to decline cigarette smoking and snus use in the future [8].

The digital game-based intervention significantly improved e-cigarette knowledge (Cohen’s *d* = 0.946; *p* < 0.001), reduced the likelihood of e-cigarette use in the coming year (Cohen’s *d* = 1.274; *p* = 0.039), and increased the perceived harm of e-cigarettes (Cohen’s *d* = 0.530; *p* = 0.002) [39]. However, it was not effective in altering attitudes toward e-cigarettes, increasing wariness surrounding e-cigarettes, or modifying perceived social norms regarding e-cigarette use [39]. Similarly, the digital game-based intervention significantly improved e-cigarette knowledge (Wilk’s λ = 0.658; *p* < 0.001; $\eta_{p}$^2^ = 0.342: large), nicotine addiction knowledge (Wilk’s λ = 0.865; *p* < 0.001; $\eta_{p}$^2^ = 0.135: moderate), perceived addictiveness of e-cigarettes (Wilk’s λ = 0.955; *p* = 0.043; $\eta_{p}$^2^ = 0.045: small), perceptions of e-cigarette harm (Wilk’s λ = 0.914; *p* = 0.001; $\eta_{p}$^2^ = 0.086: moderate), and social perceptions about e-cigarette use (Wilk’s λ = 0.973; *p* = 0.028; $\eta_{p}$^2^ = 0.027: small) [40]. Additionally, the intervention showed significant improvements in players’ knowledge about both e-cigarettes (*t* = 4.70; *p* < 0.001) and other tobacco products (*t* = 4.27; *p* < 0.001), risk perceptions related to e-cigarettes (*t* = 3.49; *p* < 0.001), and risk perceptions related to cigarettes (*t* = 2.74; *p* < 0.01) [40], along with negative health beliefs pertaining to e-cigarettes (*t* = 2.56; *p* < 0.05) and other tobacco products (*t* = 2.74; *p* < 0.05) [43].

Regarding attitudes toward various drugs and substances, the digital game-based interventions significantly increased student knowledge (Cohen’s *d* = 0.28; *p* = 0.02) [16]. Similarly, there was a significant increase in mean knowledge scores about substances (intervention mean difference [*MD*] 4.64 vs. control *MD* 0.47; *p* < 0.0001), and total attitude scores toward substances in the intervention group were also significant (intervention *MD*, 2.61 vs. control *MD*, −47.86; *p* < 0.0001) [17]. Furthermore, the intervention led to a significant development of more negative attitudes toward substances in the intervention group (*p* < 0.0001) [17]. Additionally, players who scored higher in the game had significantly greater knowledge of substance use at both the 3-month (high scoring *M* = 6.72 vs. low scoring *M* = 4.74; *t*(102) = −5.11; *p* = 0.001) and 6-month follow-ups (high scoring *M* = 6.56 vs. low scoring *M* = 4.78; *t*(102) = −4.57; *p* = 0.001) [49].

Regarding attitudes toward opioids, the digital game-based intervention significantly improved female knowledge about opioid safe storage (female *M*, 0.12 vs. male *M*, 0.04; *p* = 0.05), male knowledge about opioid misuse behavior (female *M*, 0.05 vs. male *M*, 0.14; *p* = 0.04), none-white people’s perceived knowledge of opioids (non-white *M*, 1.10 vs. white *M*, 0.75; *p* = 0.03), and older grades’s knowledge of opioids (correlation coefficient (*CC*) = −0.23, 95% *CI*, −0.40 to −0.05; *p* = 0.01) [5]. It also significantly enhanced perceived opioid knowledge (*M* = 1.08; *p* < 0.001), behavioral intent (*M* = 0.26; *p* < 0.001), safe storage knowledge (*M* = 0.12; *p* < 0.001), disposal knowledge (*M* = 0.10; *p* = 0.006), and knowledge about misuse behavior (*M* = 0.05; *p* = 0.002) [41]. Additionally, significant increases in knowledge were observed from the pre- to post-tests for both the control group (*β* = 4.45; 95% *CI*, 2.90–6.01; *p* < 0.001) and the intervention group (*β* = 6.90; 95% *CI*, 5.34–8.45; *p* < 0.001) [47]. The digital game-based intervention was also effective in increasing knowledge of opioids (e.g., “I know what opioids are”, *MD* = −1.22; *p* < 0.004), while the perception that prescription drugs are safer than illegal drugs decreased (e.g., “Prescription drugs are safer to use than illegal substances because doctors prescribe them.” *MD* = 2.14; *p* < 0.005) [1].

##### Positive Development

Of the 14 studies that included a positive development component, 35.7% (*n* = 5) reported their overall effectiveness as effective [14,17,20,25,41], 50.0% (*n* = 7) as partially effective [5,8,15,16,39,40,44], and 14.3% (*n* = 2) as not effective [21,22]. However, for positive development specifically, 57.1% (*n* = 8) showed significant differences and another 42.9% (*n* = 6) did not yield any significant findings. Some of these improvements were found in peer resistance self-efficacy (e.g., *F* = 4.21; *p* < 0.05 at post-test) [15], smoking or snus refusal efficacy (e.g., between baseline and follow-up, *p* < 0.001, between post-test and follow-up, *p* < 0.05) [8], refusal skills (*χ^2^* = 4.90; *p* < 0.05) [16], opioid safety self-efficacy between the groups (e.g., intervention *M*, 0.74 vs. control *M*, 0.30; *p* = 0.01 in [5], *M* = 0.35; *p* < 0.001 in [41]), empathy concern between pre- and post-tests (*MD* = 4.34; *p* < 0.001) [25], and fantasy dimension (*MD* = 3.13; *p* = 0.012) [25]. Studies also indicated that there were no significant differences in areas, such as opioid use self-efficacy [5] or self-efficacy to refuse e-cigarettes [39,40].

Fernandes et al. found greater improvement in cognitive reappraisal skills (*M* = 1.67; *p* = 0.001 and least squares means [LSM] difference, 1.33; 95% *CI*, 0.38–2.27; *p* < 0.01) and questions regarding their beliefs/attitudes towards the importance of changing how one thinks (*M* = 1.33; *p* = 0.01) in adolescents in the intervention group [20]. Greater improvement in cognitive reappraisal was noted in 9th graders compared to 10th graders (LSM difference, 1.63; *p* < 0.01) or 12th graders (LSM difference, 1.73; *p* = 0.02), and 11th graders exhibited greater improvement than 10th graders (LSM difference 1.33; *p* = 0.04) [20]. Female Hispanic/Latinx youths exhibited greater improvement in beliefs/attitudes compared to male Hispanic/Latinx youths (1.89 vs. 0.24; *p* = 0.03) [20]. Boendermaker et al. also reported a significant improvement in behavioral control (measured with SSRT) in the intervention group ($\eta_{p}$^2^ = 0.365; *p* < 0.001) [14].

#### 3.6.3. Facilitator or Barriers Affecting Program’s Effectiveness

Out of the 26 studies, only eight reported facilitators influencing the program’s effectiveness. Among these, five studies identified specific types of digital games as facilitators in themselves: two focused on video games, one on augmented reality cyber–physical games, one on serious games, and one on a board game. Additionally, one study identified each of the following as facilitators: activities directly related to real life, educational interventions, and eHealth interventions. However, none of the eight studies examined the statistical impact of these facilitators.

For barriers affecting the program’s effectiveness, only three studies reported content-related barriers in the game-based interventions: “video content not exactly parallel to the game”, “single-user scenarios”, and “conducted in a classroom context”. Additionally, 13 studies identified barriers related to study design and statistical issues. These included the short timeframe or follow-up duration (*n* = 6), small sample size (*n* = 5), lack of control groups (*n* = 3), high dropout rates (*n* = 2), insufficient power to detect effects (*n* = 2), reliance on self-reports instead of behavioral outcomes (*n* = 2), inclusion of only specific genders (*n* = 1) and specific ages (*n* = 1), absence of follow-up (*n* = 1), varying degrees of program fidelity (*n* = 1), primarily quantitative data (*n* = 1), lack of post-game discussion (*n* = 1), low-risk demographics (*n* = 1), a limited number of assessment items (*n* = 1), and exclusion of some data points in calculations (*n* = 1). Although these barriers were noted, none of the studies examined the statistical impact of these factors on the effectiveness of game-based interventions.

## 4. Discussion

This study aimed to identify and summarize gaps in the published literature on game-based digital interventions for substance use and positive development for adolescents through a systematic review. To our knowledge, this study is among the first to systematically review the effectiveness of game-based digital interventions for adolescent substance use and positive development. This study’s strengths include its inclusion of various types of substance use, as well as attitudes, knowledge, and perceptions related to substance use, alongside multiple aspects of positive development. Furthermore, this review encompasses a range of digital games, including VR and AR formats, across prevention, treatment, and assessment contexts. This comprehensive approach offers valuable insights into the impact of game-based digital interventions on adolescent substance use and positive development. Additionally, this study examines facilitators and barriers affecting the effectiveness of these interventions, providing a useful foundation for future research.

*Study Characteristics.* The publication years of the 26 studies ranged from 2009 to 2024, with the majority conducted in Western countries, particularly the United States (57.7%). The substantial lack of diversity in the study settings highlights the need to develop and assess the effectiveness of game-based digital interventions in non-Western regions, such as Asian countries, where culturally tailored programs may be more effective. Asian countries, including Korea and Vietnam, have unique digital gaming cultures that are closely connected to social engagement for many youths and adults [50,51]. These cultural differences suggest that intervention approaches successful in Western contexts may not fully translate to Asian populations, necessitating culturally sensitive adaptations. Furthermore, barriers related to cultural diversity, feasibility, and socio–cultural adaptability should be addressed in future interventions [7].

*Methodological Considerations.* Of the studies, 57.7% employed RCTs, while the remaining included 19.2% quasi-experimental designs and 23.1% pre-experimental studies. Regarding sampling methods, convenience sampling was used in 46.2% of the studies, highlighting potential methodological concerns given that nearly half of the studies used a non-probability sampling method despite using RCT designs. These findings underscore the necessity for more rigorous research designs, such as RCT combined with probability sampling methods, to enhance methodological robustness. On the other hand, Vajawat et al. noted that the rapid advancement of technology may render game-based interventions outdated before they are systematically tested and validated [7]. Therefore, future research should prioritize well-designed studies that can adapt swiftly to technological changes.

*Program Types and Digital Game Platforms.* Of the 26 papers reviewed, 57.7% focused on prevention programs, 30.8% on treatment programs, and 11.5% on assessment programs. This finding differs slightly from prior research on game-based interventions for substance use treatment as there were one in six studies on prevention interventions, seven in twenty-eight studies on early interventions, and two in five on treatment interventions [52]. The types of digital games varied, with 19.2% being mobile games, 15.4% utilizing virtual and augmented reality, 15.4% web-based games, 15.4% video games, 11.5% computer-based games, and 11.5% tablet-based games. The diversity of game types (e.g., serious games, social games, educational games, and gamification-based applications) demonstrated the adaptability of game-based interventions. These interventions could be particularly effective for adolescents due to their cost-effectiveness, accessibility, interactivity, and anonymity [48], which might appeal more than traditional non-interactive or lecture-based programs. Scholten et al. also found that game-based interventions could effectively engage hard-to-reach youth, such as those out of school [46], underscoring their potential for broad applications in schools and communities to increase participation and effectiveness.

*Focus Areas and Substance Use Targets.* Approximately one-third (30.8%) of the studies targeted substance use, 76.9% focused on attitudes toward substance use, and 53.8% addressed positive development. Among the studies addressing substance use, 50% focused on alcohol while 25% targeted cigarettes and opioids, respectively. This is consistent with previous studies that reported nearly half of the digital-based interventions on substance use, including serious games, were targeting alcohol use, followed by other substances/drugs such as tobacco/nicotine, cannabis, opioids, ketamine, and others [52,53]. Regarding attitudes toward substance use, 25% examined attitudes toward cigarettes, 20% targeted attitudes toward alcohol, another 20% focused on attitudes toward opioids, 15% explored attitudes toward various drugs, 10% assessed attitudes toward e-cigarettes, and 5% addressed attitudes toward both cigarettes and e-cigarettes, with another 5% focusing on general substance use. Despite the wide range of substances addressed, most studies focused primarily on alcohol, tobacco, and opioids. This suggests a need for interventions targeting the co-use of multiple substances and other illicit drugs. 

*Positive Development Focus.* Of the 14 studies examining positive adolescent development, 57.1% focused on self-efficacy while 7.1% targeted social competence, behavioral competence, and academic achievement, respectively. Furthermore, 21.4% explored multiple dimensions of positive development, with one study examining empathy and prosocial behavior; another targeting attitudes, modeling, social norms, perceived pressure, and self-efficacy; and the third focusing on cognitive reappraisal skills, beliefs/attitudes, self-efficacy, and emotional self-efficacy. This finding aligns with Martínez-Miranda and Espinosa-Curiel, who found that many serious games focus on self-efficacy as a primary outcome [53]. However, in this review, more than half of the studies focus exclusively on self-efficacy. Positive development is multidimensional, and future research should consider incorporating additional aspects, such as socio–emotional learning, into game-based interventions to support a more holistic approach to positive adolescent development. This could promote self-esteem and emotional balance and reduce emotional problems [54].

*Effectiveness of Game-Based Interventions.* Among the studies targeting substance use, 75% reported statistically significant effectiveness for game-based interventions, all of which targeted cigarette smoking or alcohol consumption. Of these, 50% were deemed effective in reducing substance use, 16.7% were partially effective, and 33.3% concluded that they were not effective. Studies on attitudes toward substance use reported 65% statistical effectiveness, with the rest partially effective (25%) or not significant (10%). Among the studies addressing positive development, 35.7% were effective, 50% partially effective, and 14.3% were not effective. These mixed results indicate that while game-based interventions show promise, future research is needed to investigate factors contributing to variability in effectiveness.

*Facilitators and Barriers.* Only 30.8% of the studies reported facilitators influencing program effectiveness, with 62.5% identifying specific types of digital games (e.g., video games, augmented reality cyber–physical games, serious games, and board games). Real-life activities, educational content, and eHealth interventions as additional facilitators in some studies. Conversely, only 11.5% of the studies reported content-related barriers affecting the effectiveness of game-based interventions, citing issues such as “video content not aligning with the game”, “single-user scenarios”, and “conducted in a classroom context”. However, none of the eight studies assessed the statistical impact of these facilitators and barriers, indicating a need for future research to address these facilitators and barriers in the development process. Doing so not only enhances the accessibility and widespread use of game-based interventions but also improves the overall effectiveness of these programs. For instance, Aneni et al. explained that the game-based intervention should reflect youth preferences over video game details, including mechanics, esthetics, and storyline content, which oftentimes serve as a main facilitator in the game-based intervention programs [55].

*Potential Risks and Practical Challenges.* No studies have explored the potential side effects of digital game-based intervention programs. Existing reports suggest that these interventions could lead to unintended consequences, such as gaming addiction, particularly among adolescents with conditions like externalizing spectrum disorders or substance use disorders [7,56]. Similarly, Montanaro et al. [49] explained that more time spent in the intervention lead to greater behavioral changes, these effects often arise from the mastery of the game itself. Practical issues, such as limited network bandwidth, inconsistent internet access, and inadequate infrastructure in remote and rural areas, can also disrupt these interventions, challenging their availability and acceptability [7]. Additional concerns include unauthorized access, data theft, abuse or misuse, and the potential for cybercrime. The absence of established guidelines, legislation, or authorization protocols for the use of game-based interventions in mental health care further complicates their implementation [7]. Accessibility challenges are also pronounced for socially disadvantaged and vulnerable populations [7]. Despite these risks, none of the studies in this review addressed them, underscoring the need for future research to examine these potential side effects and develop mitigation strategies. Moreover, practitioners, including program facilitators, should receive thorough training in-game time management, cybersecurity practices, and ethical guidelines for gaming to ensure that participants can engage safely and effectively.

### Limitations

This systematic review has several limitations. First, the selection of studies from four databases—PubMed, PsycINFO, Scopus, and CINAHL—along with the keywords used may not have captured all relevant research. Additionally, we did not perform technical evaluations of the game-based digital interventions, indicating the need for future research to explore technical aspects in greater detail. The variety of study types and the limited number of studies addressing different target variables also made it challenging to conduct a meta-analysis. However, as more research becomes available, performing meta-analyses will be increasingly valuable. 

## 5. Conclusions

This systematic review examines game-based digital interventions targeting substance use, attitudes toward substance use, and positive development of the adolescent population. It provides valuable insights into the most recent trends in game-based digital interventions and lays a solid foundation for leveraging digital game technology to address substance use problems and promote positive development. This review highlights several key implications for the development and evaluation of game-based digital interventions: the need for more rigorous experimental research designs, extending the focus to include a wider variety of substance types and aspects of positive development, conducting active research in diverse countries with respect to cultural relevance and sensitivity, assessing the impact of facilitators and barriers on program effectiveness, and examining potential side effects while developing strategies to mitigate these concerns during implementation. While the review offers valuable insights, it has limitations, including potential gaps in the search process, the absence of technical evaluations of the game-based digital interventions, and difficulties in conducting a meta-analysis due to the variety of study types and the limited number of studies. Future studies can build upon this research by addressing these limitations, which could further improve intervention strategies and effectiveness in combating substance use and promoting positive development.

## Figures and Tables

**Table 1:** PICO * and other inclusion and exclusion criteria.

	Inclusion Criteria	Exclusion Criteria
Population	Adolescents only	Not applicable
Intervention	Any type of digital game-based intervention for substance use or positive development	Not applicable
Control	Not applicable	Not applicable
Outcomes	All reported outcomes related to substance use or positive development	Not applicable
Study design	Experimental studies: RCTs and non-RCTs	Qualitative research, observational studies: case-control studies and cohort studies.
Language	English only	Not applicable
Information	Effect size data provided (e.g., odds ratio, 95% CI, or other effect size values)	Effect size data not provided
Study quality	Rated as “fair” or “good” based on the National Institute of Health (NIH) quality assessment tool	Rated as “poor” based on the NIH quality assessment tool
Publication type	Peer-reviewed articles	Master’s theses or doctoral dissertations, commentary and editorials, review papers (systematic review and meta-analysis)
Full text	Full texts available	Full texts unavailable

* PICO: population, intervention, comparison, and outcome.

**Table 2:** Summary of intervention effectiveness ^a^.

Results of Intervention	Number of Studies (Reference Numbers in Brackets)		
	Substance Use	Attitudes Toward Substance Use	Positive Development
Significant	4 [4,22,24,38]	13 [4,5,16,17,23,38,41,42,43,44,45,48,49]	5 [14,17,20,25,41]
Partially significant	1 [46]	5 [1,22,39,40,47]	7 [5,8,15,16,39,40,44]
Not significant	2 [5,14]	2 [8,21]	2 [21,22]

^a^ The number of trials in each cell does not add up to the total number of trials, as many appear more than once.

## Data Availability

The original contributions presented in the study are included in the article, further inquiries can be directed to the corresponding author.

## Appendix A

**Table A1:** Main findings.

Author (Year), Game-Interventions Name	Study Type	Main Substance	Main Attitude	Main Positive Development	Type of Intervention/ Game	Sample Size	Therapy or Theory	Duration/Session	Effectiveness—Descriptive Analysis	Effectiveness—Comparative Analysis	Facilitator	Barrier
Norris et al. (2013), **DRAMA-RAMA**, Florida, USA [15]	Randomized controlled trial	N/A	N/A	Self-efficacy	Treatment	41	Communication competence model (CCM)	15 min/5	- 3.33 on a scale of 1–4 (Liked the game) - 90.5% (Felt it real) - 71.4% (Deeply involved with it)	- Increased peer resistance self-efficacy (*F* = 4.21; *p* < 0.05)	N/A	N/A
Scholten et al. (2019), **HitnRun**, Netherlands [46]	Randomized controlled trial	Cigarette	N/A	N/A	Treatment	144	N/A	2–5 min/2	- Rated the game intervention more favorably compared to control group (intervention, *M* 15.21 vs. control, *M* 13.49, *t*(124) = −2.50; *p* = 0.014)	- Increased cigarette cessation time (*F* (2, 138) = 79.50; *p* < 0.001, $\eta_{p}$^2^ = 0.54)	N/A	N/A
Weser et al. (2021), **Invite Only VR**, USA [39]	One-group pretest–post-test design (pre-experimental)	N/A	Knowledge, likelihood of use, perceived harm, attitudes towards e-cigarettes	Self-efficacy	Prevention	47	Theory of Planned Behavior (TPB); Social Cognitive Theory (SCT)	46.5 min/1–2	- 3 on a scale of 1–4 (Enjoyed playing the game)	- Increased e-cigarette knowledge (Cohen’s *d* = 0.946; *p* < 0.001) - Decreased likelihood of using e-cigarettes in coming year (Cohen’s *d* = 1.274; *p* = 0.039) - Increased perceived harm of e-cigarettes (Cohen’s *d =* 0.530; *p* = 0.002)	N/A	N/A
Nyman et al. (2024), **Fume**, Finland [8]	Randomized controlled trial	N/A	Motivation to decline smoking and snus use	Self-efficacy	Prevention	781	Self-Efficacy Theory (SET)	15–30 min/2	N/A	- Increased smoking refusal self-efficacy between baseline and follow-up (*p* = 0.001) and between post-test and follow-up (*p* < 0.05)	N/A	N/A
Ho et al. (2021), **An Internet Quiz Game Intervention**, Hong Kong [4]	Randomized controlled trial	Alcohol	Perceived social norms, alcohol-related knowledge	N/A	Treatment	7792	N/A	4 weeks/N/A	N/A	- Decreased drinking behavior (*RR* = 0.79, 95% *CI* = 0.68–0.92; *p* = 0.003) - Decreased alcohol consumption (*β* = −0.06, 95% *CI* = −0.11–−0.01; *p* = 0.02) - Decreased alcohol-related troubles (*β* = 0.62, 95% *CI* = 0.61–0.62; *p* < 0.001) - Increased perception of parents and peers to be more anti-alcohol (*β* = 0.06, 95% *CI* = 0.00–0.12; *p* < 0.05) - Increased alcohol-related knowledge (*β* = 0.18, 95% *CI* = −0.01–0.36; *p* = 0.07)	N/A	N/A
Parisod et al. (2018), **Fume**, Finland [44]	Randomized controlled trial	N/A	Smoking outcome expectation, attitudes towards tobacco, tobacco-use motives, motivation to decline tobacco use in the future, knowledge about tobacco) outcome	Self-efficacy	Prevention	151	N/A	2 weeks/4	- 25.4% (Rated “very nice”) - 52.5% (Rated “nice”)	- Change in positive (*p* = 0.002) and negative (*p* = 0.02) smoking outcome expectations - Change in attitudes towards cigarette smoking (*p* = 0.01) within the group - Raised more interest compared to website offered to control group (70.0% in intervention group vs. 27.7% in control group; *p* < 0.001)	N/A	N/A
Yap et al. (2020), **Cognitive-Behavioral Game Design-Based Mobile Game**, Philippines [38]	Randomized controlled trial	Alcohol	Attitudes toward drinking and alcoholism, alcohol knowledge	N/A	Treatment	140	The Cognitive Behavioral Game Design Theory (CBGDT)	20–40 min/1	N/A	- Increased knowledge (Cohen’s *d* = 2.54 in intervention group vs. Cohen’s *d* = 0.89 in control group; *p* = 0.00)	N/A	Video content was not exactly parallel to that of the mobile game
Rose & Unni (2020), **I’M HAPPY**, Utah, USA [16]	Randomized controlled trial	N/A	Knowledge about prescription and OTC drugs	Social competence	Prevention	34	Theory of Gamified Learning (TGL); Social Learning Theory (SLT); Theory of Reasoned Action (TRA)	20–40 min/1	- 79% (Learned new things) - 88% (Found it enjoyable) - 97% (Found it easy to use)	- Increased knowledge (Cohen’s *d =* 0.28; *p* = 0.02) - Increased proportion of students finding refusal skills to be “very helpful”: “naming the problems” (χ^2^ = 4.90; *p* = 0.03)	All activities relate directly to real life	N/A
Abraham et al. (2021), **The SG MedSMART: Adventures in PharmaCity**, USA [5]	One-group pretest–post-test design (pre-experimental)	Opioid	Naloxone/opioid/Narcan knowledge, behavioral intent, knowledge about misuse behavior/misuse harm/safe storage/safe disposal, perceived knowledge	Self-efficacy (MUSE, opioid safety)	Prevention	117	N/A	30 min/1	N/A	- Females’ increased knowledge about safe storage (female *M,* 0.12 vs. male, *M* 0.04; *p* = 0.05) - Males’ increased knowledge about misuse behavior (female, *M* 0.05 vs. male, *M* 0.14; *p* = 0.04) - Non-white or Hispanics’ increased perceived knowledge (non-white, *M* 1.10 vs. white, *M* 0.75; *p* = 0.03) - Older grades’ increased opioid knowledge (*CC =* −0.23, 95% *CI =* −0.40–−0.05; *p* = 0.01) - Increased self-efficacy (opioid safety) (intevention, *M* 0.74 vs. control, *M* 0.30; *p* = 0.01)	N/A	N/A
Boendermaker et al. (2017), **The Fling**, Netherlands [14]	Randomized controlled trial	Alcohol	N/A	Behavioral competence	Treatment	185	N/A	10–15 min/4	N/A	- Increased behavioral control ($\eta_{p}$^2^ = 0.365; *p <* 0.001)	N/A	SSRT was calculated making mild exclusions
Weser et al. (2021), **Invite Only VR**, USA [40]	Randomized controlled trial	N/A	E-cigarette knowledge, nicotine addiction knowledge, perceived addictiveness of e-cigarettes, perceived likelihood of using e-cigarettes, perceptions of harm, social approval of e-cigarettes, e-cigarette social perceptions	Self-efficacy	Prevention	279	TPB; SCT	40 min/2–4	- 3.08 on a scale of 1–4 (Enjoyed the game) - 2.91 on a scale of 1–4 (Felt “being there”)	- Increased e-cigarette knowledge (Wilk’s λ = 0.658; *p* < 0.001, $\eta_{p}$^2^ = 0.342 (large)) - Increased nicotine addiction knowledge (Wilk’s λ = 0.865; *p* < 0.001, $\eta_{p}$^2^ = 0.135 (moderate)) - Increased perceived addictiveness of e-cigarettes (Wilk’s λ = 0.955; *p* = 0.043, $\eta_{p}$^2^ = 0.045 (small)) - Increased perceptions of e-cigarette harm (Wilk’s λ = 0.914; *p* = 0.001, $\eta_{p}$^2^ = 0.086 (moderate)) - Increased Social perceptions about e-cigarette use (Wilk’s λ = 0.973; *p* = 0.028, $\eta_{p}$^2^ = 0.027 (small))	N/A	N/A
Rath et al. (2015), **Flavor Monster by The Truth Campaign**, USA [45]	One-group pretest–post-test design (pre-experimental)	N/A	Attitudes about tobacco products and the tobacco industry	N/A	Assessment	693	TPB	270 min/N/A	N/A	- Change in attitudes about tobacco products and the tobacco industry at 3-month follow-up (*MD* = 0.6, Cohen’s *d* = −0.36; *p <* 0.0001)	N/A	N/A
Abraham et al. (2023), **The SG MedSMART: Adventures in PharmaCity**, USA [41]	One-group pretest–post-test design (pre-experimental)	N/A	Knowledge about opioids	Self-efficacy	Prevention	60	SCT; TPB	30 min/1	N/A	- Increased opioid safety self-efficacy (*M* = 0.35, *SD* = 0.60; *p* < 0.001) - Increased perceived knowledge (*M* = 1.08; *p* < 0.001) - Increased disposal knowledge (*M* = 0.10; *p* = 0.006) - Increased knowledge about misuse behavior (*M* = 0.05; *p* = 0.002)	N/A	N/A
Abroms et al. (2015), **Recovery Warrior**, Washington, D.C., USA [21]	Quasi-experimental	Opioid	Reduce cravings (opioids)	Self-efficacy	Treatment	6	SCT; Reinforcement Theory of Motivation (RTM)	30 min/4	- Based on urine analysis, four out of nine participants (44.44%) remained abstinent from opioid use	N/A	N/A	- Small sample - No control group
Fernandes et al. (2023), **empoweree**, Connecticut, USA [20]	Randomized controlled trial	N/A	N/A	Cognitive reappraisal skills	Assessment	98	Cognitive Behavioral Therapy (CBT); Game Therapy (GT)	13.04 min/1	- From −3 to 3, participants reported the game was supportive (*M* = 1.1), easy (*M* = 1.6), efficient (*M* = 1.1), and clear (*M* = 1.7)	- empowerED group greater improvement in cognitive reappraisal (*M* = 1.67; *p* < 0.001) and beliefs/attitudes (*M* = 1.33; *p* = 0.01) - empowerED group, cognitive reappraisal improvement compared to control group (least squares means [LSM] difference 1.33, 95% *CI*, 0.38–2.27; *p* < 0.01) - Greater improvement in cognitive reappraisal in 9th graders compared to 10th graders (LSM Difference = 1.63; *p* = 0.01) or 12th graders (LSM Difference = 1.73; *p* = 0.02), and 11th graders exhibited greater improvement than 10th graders (LSM Difference = 1.33; *p* = 0.04) - Female Hispanic/Latinx youths exhibited greater improvement in beliefs/attitudes compared to male Hispanic/Latinx youths (*MF* = 1.89, *MM* = −0.24; *p* = 0.03)	N/A	N/A
Gilliam et al. (2019), **Smoke Stacks**, Illinois, USA [23]	Quasi-experimental	N/A	Knowledge and attitudes toward tobacco companies	N/A	Prevention	67	TPB	60 min/1	N/A	- Increased perceived knowledge of health effects of tobacco (Pretest, *M* = 2.81, 95% *CI*: 2.61–3.01 vs. Post-test, *M* = 3.31, 95% *CI*: 3.15–3.47; *t* = −4.79; *p* < 0.001)	N/A	- Small sample - No power calculation - Low risk demographic
Hieftje et al. (2019)**, smokeSCREEN**, Connecticut, USA [42]	Quasi-experimental	N/A	Harmful to health (tobacco)	N/A	Prevention	560	SCT; TPB	88.2 min	- Females more likely (*OR* ¼ 1.84 (1.27–2.66)) to enjoy playing the game than males and more likely (*OR* ¼ 1.59 (1.06–2.38)) to state that they learned something new - Males were more likely (*OR* ¼ 4.11 (1.06–15.88)) to feel game was challenging than participants that preferred to self-describe	- Significant differences in the proportion of correct answers were observed for questions related to beliefs about the health harms of cigarettes and e-cigarettes, their danger, and the potential for teen addiction, questions assessing knowledge of nicotine content, tobacco marketing toward teens, the impact on teen brain development, and the likelihood of smoking as an adult (all *p* < 0.0001)	N/A	- Did not have 14–16 year-olds enroll - 33% did not complete post-survey
Jander et al. (2016), **Alcohol Alert**, Netherlands [22]	Randomized Controlled Trial	Alcohol	Intention to decrease alcohol use	Self-efficacy	Treatment	2649	I-Change Model	N/A/3	N/A	- Intervention decreased binge drinking both for 15-year-olds (after 3 sessions: *OR* = 0.22, 95% *CI* = 0.09–0.54; *p* = 0.04), and for 16-year-olds after three sessions (*OR* = 0.37, 95% *CI* = 0.16–0.87; *p* = 0.02) - Excessive drinking revealed a significant interaction effect between condition and educational level (*OR* 2.37, 95% *CI* 0.98–5.73; *p* = 0.05) for adolescents that adhered to at least 1 session - Parent participation in intervention; children reported less binge drinking in previous 30 days (*p* = 0.04) - Higher educational level (*p* < 0.001), lower age (*p* < 0.001), and being Protestant (*p* = 0.03), Muslim (*p* < 0.001), or a member of another religion (*p* = 0.03) were significant protective determinants of binge drinking - Protective determinants of excessive drinking: female (*p* < 0.001), a higher educational level (*p =* 0.01), and being younger (*p* < 0.001).	N/A	- Low adherence rates - Outcome based on self-report - Attrition rates
Lopez-Faican et al. (2023), **EmpathyAR**, Spain [25]	Randomized Controlled Trial	N/A	N/A	Empathy	Assessment	30	Flow Theory (FT)	4 days/4	N/A	- Experimental group’s empathic concern (*MD* = 4.34; *p* < 0.001) and fantasy dimension (*MD* = 3.13; *p* < 0.012)	Use of geolocation	- Single-user scenarios - Short-term evaluation - Small sample
Montanaro et al. (2015), **PlayForward: Elm City Stories**, Connecticut, USA [49]	Randomized Controlled trial	N/A	Knowledge of substance use	N/A	Prevention	144	SCT; Prospect Theory (PT); Health Behavior Theory (HBT)	436.2 min/N/A	N/A	- Higher game score had significantly greater knowledge of substance use at both the 3-month (high scoring: *M* = 6.72; low scoring: *M* = 4.74; *t*(102) = −5.11; *p* = 0.001) and 6-month follow-ups (high scoring: *M* = 6.56; low scoring: *M* = 4.78; *t*(102) = −4.57; *p* = 0.001)	N/A	- Small number of items to assess substance use knowledge
Oliveto et al. (2022), **SafeUse**, Arkansas, USA [1]	Mixed method	N/A	Knowledge and perception of opioid use	N/A	Prevention	14	SCT; Information–Motivation–Behavioral Skills model	42.7 min/4.7	- Moderate to high levels of enjoyment for the game, specific statistical data not provided	- Increased knowledge of opioids (“I know what opioids are.” *MD* = −1.22; *p* < 0.004) - Decreased perception that prescription drugs are safer than illegal drugs (“Prescription drugs are safer to use than illegal substances because doctors prescribe them.” *MD* = 2.14; *p* < 0.005)	N/A	- Small sample
Pentz et al. (2019), **PlayForward: smokeScreen**, California, USA [43]	Quasi-experimental with cross-replication	N/A	Knowledge of tobacco products	N/A	Prevention	80	N/A	60 min/4	N/A	- Increased knowledge of e-cigarettes and other tobacco products (*t* = 4.70; *p* < 0.001; *t* = 4.27; *p* < 0.001, respectively) - Increased risk perceptions related to e-cigarettes (*t* = 3.49; *p* < 0.001) and cigarettes (*t* = 2.74; *p* < 0.01) - Negative health beliefs pertaining to e-cigarettes (*t* = 2.56; *p* < 0.05) and other tobacco products (*t* = 2.74; *p* < 0.05)	N/A	- No control group - Small sample size - Short time frame
Stapinski et al. (2018), **Pure Rush**, Australia [47]	Randomized controlled trial	N/A	Future intention to use illicit drugs	N/A	Prevention	281	N/A	10 min/1	- 91% expressed enjoyment of the game designed for attitudes toward illicit drugs - 88% rated it as superior to a traditional booklet - 92% felt it made the lesson more engaging	- Increased knowledge pre- to post-test for both the control group (*β* = 4.45, 95% *CI*: 2.90–6.01; *p* < 0.001) and intervention group (*β* = 6.90, 95% *CI*: 5.34–8.45; *p* < 0.001)	N/A	- No follow-up - Classroom context limiting intervention time
Stein-Seroussi et al. (2009), **ACTION** (**Adolescent Cessation of Tobacco: Independent of Nicotine)**, North Carolina, USA [24]	Randomized Controlled Trial	Tobacco	N/A	N/A	Treatment	261	Stages of Change (SOC), Psychosocial Theory	50 min/6	N/A	- Group differences significant at 90-day follow-up for 3-day abstinence with verification (*β* (*SE*) = 1.49 (0.72); *p* = 0.048, *OR* (*CI*) = 4.46 (1.02–19.52)) and 7-day abstinence with 3-day verification (*β* (*SE*) = 1.49 (0.72); *p* = 0.049; *OR* (CI) = 4.44 (1.01–19.49))	N/A	- Lack of power to detect effects - Varying degrees of program fidelity
Taghipour et al. (2023), **N/A**, Iran [17]	Quasi-experimental with control	N/A	Knowledge of substance abuse	Academic achievement	Prevention	114	N/A	N/A	N/A	- Increased mean knowledge scores about substances (intervention, *MD* 4.64 vs. control, *MD* 0.47; *p* < 0.0001) - Increased total attitude scores toward substances (control group: intervention, *MD* 2.61 vs. control, *MD* −47.86; *p* < 0.0001) - Significant development of more negative attitudes toward substances in the intervention group (*p* < 0.0001)	N/A	- Short duration follow-up - Only males
Willmott et al. (2019), **Game On: Know Alcohol**, Australia [48]	Pretest–post-test design (pre-experimental)	N/A	Psychosocial determinants of binge drinking	N/A	Prevention	303	TRA	N/A	N/A	- Increased gameplay, more positive attitudes toward binge drinking (*β* = 0.12; *p* < 0.05) - Higher game scores associated with more negative attitudes toward binge drinking (*β* = −0.25; *p* < 0.001)	N/A	- 1-day school-based study - Short interval between pre and post-measures

## References

[1] Oliveto A.H., Wright P., Kumar N., Gokarakonda S., Fischer-Laycock I., Williams J., Thompson R.G. (2022). Acceptability of a game-based intervention to prevent adolescent prescription opioid misuse. Games Health J..

[2] Wouters P., Van Nimwegen C., Van Oostendorp H., Van Der Spek E.D. (2013). A meta-analysis of the cognitive and motivational effects of serious games. J. Educ. Psychol..

[3] Zhan J., Liu C., Wang Z., Cai Z., He J. (2024). Effects of game-based digital interventions for mental disorders: A meta-analysis. J. Affect. Disord..

[4] Ho F.K., Tung K.T.S., Wong R.S., Chan K.L., Wong W.H.S., Ho S.Y., Lam T.H., Mirpuri S., Van Voorhees B., Fu K.W. (2021). An internet quiz game intervention for adolescent alcohol drinking: A clustered RCT. Pediatrics.

[5] Abraham O., Rosenberger C., Tierney K., Birstler J. (2021). Investigating the use of a serious game to improve opioid safety awareness among adolescents: Quantitative study. JMIR Serious Games.

[6] Ryan R.M., Rigby C.S., Przybylski A. (2006). The motivational pull of video games: A self-determination theory approach. Motiv. Emot..

[7] Vajawat B., Varshney P., Banerjee D. (2021). Digital gaming interventions in psychiatry: Evidence, applications and challenges. Psychiatry Res..

[8] Nyman J., Salanterä S., Pasanen M., Parisod H. (2024). Effectiveness of a digital health game intervention on early adolescent smoking refusal self-efficacy. Health Educ. Behav..

[9] Miech R.A., Johnston L.D., Patrick M.E., O’Malley P.M. Monitoring the Future National Survey Results on Drug Use, 1975–2023: Overview and Detailed Results for Secondary School Students. https://monitoringthefuture.org/results/annual-reports/.

[10] Gray K.M., Squeglia L.M. (2017). Research Review: What have we learned about adolescent substance use?. J. Child. Psychol. Psych..

[11] Lee T.Y. (2011). Construction of an integrated positive youth development conceptual framework for the prevention of the use of psychotropic drugs among adolescents. Sci. World J..

[12] Lotrean L.M., Mesters I., de Vries H. (2013). Why do Romanian junior high school students start to smoke?. Child Care Health Dev..

[13] Wang Y., Tian L., Huebner E.S. (2019). Parental control and Chinese adolescent smoking and drinking: The mediating role of refusal self-efficacy and the moderating role of sensation seeking. Child. Youth Serv. Rev..

[14] Boendermaker W.J., Veltkamp R.C., Peeters M. (2017). Training behavioral control in adolescents using a serious game. Games Health J..

[15] Norris A.E., Hughes C., Hecht M., Peragallo N., Nickerson D. (2013). Randomized trial of a peer resistance skill-building game for Hispanic early adolescent girls. Nurs. Res..

[16] Rose T.M., Unni E.J. (2020). A pilot evaluation of I’M HAPPY: An interactive module to halt abuse of prescriptions in preteens and youth. Games Health J..

[17] Taghipour E., Vizeshfar F., Zarifsanaiey N. (2023). The effect of gamification-based training on the knowledge, attitudes, and academic achievement of male adolescents in preventing substance and internet addiction. BMC Med. Educ..

[18] Trucco E.M., Hartmann S.A., Fallah-Sohy N. (2024). Charting a course for empowered adolescent substance use treatment. Clin. Psychol..

[19] dos Santos T.T., Ríos M.P., de Medeiros G.C.B.S., Mata Á.N.D.S., Silva Junior D.D.N., Guillen D.M., Piuvezam G. (2023). Gamification as a health education strategy of adolescents at school: Protocol for a systematic review and meta-analysis. PLoS ONE.

[20] Fernandes C.F., Deng Y., Tran A.H., Hieftje K.D., Boomer T.M.P., Taylor C.K., Fiellin L.E. (2023). A pilot randomized controlled trial to evaluate a cognitive behavioral videogame intervention: empowerED. Games Health J..

[21] Abroms L.C., Leavitt L.E., Van Alstyne J.M., Schindler-Ruwisch J.M., Fishman M.J., Greenberg D.A. (2015). Motion videogame for opioid relapse prevention. Games Health J..

[22] Jander A., Crutzen R., Mercken L., Candel M., de Vries H. (2016). Effects of a web-based computer-tailored game to reduce binge drinking among Dutch adolescents: A cluster randomized controlled trial. J. Med. Internet Res..

[23] Gilliam M., Hill B.J., Jaworski E., Sparrow A., Jones I.B., Jagoda P. (2019). Increasing anti-tobacco industry attitudes among youth: A pilot study of a multiplayer educational board game. Games Health J..

[24] Stein-Seroussi A., Stockton L., Brodish P., Meyer M. (2009). Randomized controlled trial of the ACTION smoking cessation curriculum in tobacco-growing communities. Addict. Behav..

[25] López-Faican L., Jaen J. (2023). Design and evaluation of an augmented reality cyberphysical game for the development of empathic abilities. Int. J. Hum.-Comput. Stud..

[26] Moore S.E., Norman R.E., Suetani S., Thomas H.J., Sly P.D., Scott J.G. (2017). Consequences of bullying victimization in childhood and adolescence: A systematic review and meta-analysis. World J. Psychiatry.

[27] Christensen J., Valentiner L.S., Petersen R.J., Landberg H. (2015). The effect of game-based interventions in rehabilitation of diabetics: A systematic review and meta-analysis. Telemed. e-Health.

[28] Cuevas-Lara C., Izquierdo M., Sáez de Asteasu M.L., Ramírez-Vélez R., Zambom-Ferraresi F., Zambom-Ferraresi F., Martínez-Velilla N. (2021). Impact of game-based interventions on health-related outcomes in hospitalized older patients: A systematic review. J. Am. Med. Dir. Assoc..

[29] Li J., Theng Y., Foo S. (2014). Game-based digital interventions for depression therapy: A systematic review and meta-analysis. Cyberpsychol. Behav. Soc. Netw..

[30] Suleiman-Martos N., García-Lara R.A., Membrive-Jiménez M.J., Pradas-Hernández L., Romero-Béjar J.L., Dominguez-Vías G., Gómez-Urquiza J.L. (2021). Effect of a game-based intervention on preoperative pain and anxiety in children: A systematic review and meta-analysis. J. Clin. Nurs..

[31] Degenhardt L., Bucello C., Calabria B., Nelson P., Roberts A., Hall W., Lynskey M., Wiessing L., The GBD illicit drug use writing group (2011). What data are available on the extent of illicit drug use and dependence globally? Results of four systematic reviews. Drug Alcohol. Depen..

[32] Substance Abuse and Mental Health Services Administration Key Substance Use and Mental Health Indicators in the United States: Results from the 2022 National Survey on Drug Use and Health. https://store.samhsa.gov/product/results-2022-national-survey-drug-use-and-health-nsduh-key-substance-use-and-mental-health.

[33] Moher D., Liberati A., Tetzlaff J., Altman D.G., PRISMA Group (2009). Preferred reporting items for systematic reviews and meta-analyses: The PRISMA statement. Ann. Intern. Med..

[34] Covidence Better Systematic Review Management. https://www.covidence.org/.

[35] NIH Study Quality Assessment Tools: National Institute of Health. https://www.nhlbi.nih.gov/health-topics/study-quality-assessment-tools.

[36] Bunting L., Davidson G., McCartan C., Hanratty J., Bywaters P., Mason W., Steils N. (2018). The Association between child maltreatment and adult poverty–a systematic review of longitudinal research. Child. Abuse Negl..

[37] McHugh M.L. (2012). Interrater reliability: The kappa statistic. Biochem. Medica.

[38] Yap A.G.H., Roy R.E.D., Lasala J.R.S., Tan D.K., Hechanova M.R., Diy W.D.A., Rodrigo M.M.T. (2020). Evaluation of a cognitive-behavioral game design-based mobile game on alcohol use for adolescents. Games Health J..

[39] Weser V.U., Duncan L.R., Pendergrass T.M., Fernandes C.S., Fiellin L.E., Hieftje K.D. (2021). A quasi-experimental test of a virtual reality game prototype for adolescent E-Cigarette prevention. Addict. Behav..

[40] Weser V.U., Duncan L.R., Sands B.E., Schartmann A., Jacobo S., François B., Hieftje K.D. (2021). Evaluation of a virtual reality e-cigarette prevention game for adolescents. Addict. Behav..

[41] Abraham O., McCarthy T.J., Zaborek J. (2023). Assessing the impact of a serious game (MedSMARxT: Adventures in PharmaCity) in improving opioid safety awareness Among Adolescents and Parents: Quantitative Study. JMIR Form. Res..

[42] Hieftje K.D., Fernandes C.-S.F., Lin I.-H., Fiellin L.E. (2021). Effectiveness of a web-based tobacco product use prevention videogame intervention on young adolescents’ beliefs and knowledge. Subst. Abus..

[43] Pentz M.A., Hieftje K.D., Pendergrass T.M., Brito S.A., Liu M., Arora T., Tindle H.A., Krishnan-Sarin S., Fiellin L.E. (2019). A videogame intervention for tobacco product use prevention in adolescents. Addict. Behav..

[44] Parisod H., Pakarinen A., Axelin A., Löyttyniemi E., Smed J., Salanterä S. (2018). Feasibility of mobile health game “Fume” in supporting tobacco-related health literacy among early adolescents: A three-armed cluster randomized design. Int. J. Med. Inform..

[45] Rath J.M., Williams V., Rubenstein R., Smith L., Vallone D. (2015). Assessing the impact of an interactive mobile game on tobacco-related attitudes and beliefs: The truth campaign’s “flavor monsters”. Games Health J..

[46] Scholten H., Luijten M., Granic I. (2019). A randomized controlled trial to test the effectiveness of a peer-based social mobile game intervention to reduce smoking in youth. Dev. Psychopathol..

[47] Stapinski L.A., Reda B., Newton N.C., Lawler S., Rodriguez D., Chapman C., Teesson M. (2018). Development and evaluation of ‘Pure Rush’: An online serious game for drug education. Drug Alcohol. Rev..

[48] Willmott T., Russell-Bennett R., Drennan J., Rundle-Thiele S. (2019). The impact of serious educational gameplay on adolescent binge drinking intentions: A theoretically grounded empirical examination. Health Educ. Behav..

[49] Montanaro E., Fiellin L.E., Fakhouri T., Kyriakides T.C., Duncan L.R. (2015). Using videogame apps to assess gains in adolescents’ substance use knowledge: New opportunities for evaluating intervention exposure and content mastery. J. Med. Internet Res..

[50] Chun J., Kim J., Lee S. (2023). Development of a cyberbullying victimization scale for adolescents in South Korea. Child. Youth Serv. Rev..

[51] McCauley B., Nguyen T.H.T., McDonald M., Wearting S. (2020). Digital gaming culture in Vietnam: An exploratory study. Leis. Stud..

[52] Monarque M., Sabetti J., Ferrari M. (2023). Digital interventions for substance use disorders in young people: Rapid review. Subst. Abuse Treat. Prev. Policy.

[53] Martínez-Miranda J., Espinosa-Curiel I.E. (2022). Serious games supporting the prevention and treatment of alcohol and drug consumption in Youth: Scoping Review. JMIR Serious Games.

[54] De la Barrera U., Monaco E., Postigo-Zegarra S., Gil-Gomez J., Montoya-Castila I. (2021). EmoTIC: Impact of a game-based social-emotional programme on adolescents. PLoS ONE.

[55] Aneni K., Fernandes C.F., Hoerner L.A., Szapary C., Boomer T.M.P., Fiellin L.E. (2023). A video game intervention to prevent opioid misuse among older adolescents: Development and preimplementation study. JMIR Serious Games.

[56] Männikkö N., Mendes L., Barbosa F., Reis L.P. (2014). Health determinants related to digital game playing: A systematic review. J. Health Sci..

